# Tree Imagery in Drawing Tests for Screening Mental Disorders: A Systematic Review and Meta‐Analysis

**DOI:** 10.1155/da/9571222

**Published:** 2026-07-11

**Authors:** Huibing Guo, Bin Feng, Tiantian Liu, Ruopeng Zhao, Huiyong Fan, Zaiquan Dong, Qiyong Gong, Taolin Chen

**Affiliations:** ^1^ Department of Radiology, Huaxi MR Research Center (HMRRC), Institute of Radiology and Medical Imaging, Psychoradiology Key Laboratory of Sichuan Province, West China Hospital of Sichuan University, Chengdu, Sichuan, China, wchscu.cn; ^2^ Department of Student Affairs Management, West China School of Medicine, Sichuan University, Chengdu, China, scu.edu.cn; ^3^ West China Hospital, Sichuan University, Chengdu, China, scu.edu.cn; ^4^ Teacher Education College, Dali University, Dali, China, dali.edu.cn; ^5^ College of Medical Technology, West China Hospital, Sichuan University, Chengdu, China, scu.edu.cn; ^6^ Institute of Education, Bohai University, Jinzhou, China, bhu.edu.cn; ^7^ Mental Health Center, West China Hospital, Sichuan University, Chengdu, China, scu.edu.cn; ^8^ Xiamen Key Lab of Psychoradiology and Neuromodulation, Department of Radiology, West China Xiamen Hospital of Sichuan University, Xiamen, Fujian, China, scu.edu.cn; ^9^ Research Unit of Psychoradiology, Chinese Academy of Medical Sciences, National Center for Mental Disorder, National Clinical Research Center for Geriatrics, State Key Laboratory of Biotherapy, West China Hospital, Sichuan University, Chengdu, Sichuan, China, scu.edu.cn

**Keywords:** affective and thought disorders, drawing test, mental disorders, meta-analytic review, systematic review, tree imagery

## Abstract

Although tree imagery in projective drawing tests is a promising nonverbal tool for screening mental disorders, its clinical utility remains constrained by inconsistent predictor selection. To address this gap, the present systematic review and meta‐analysis synthesizes the characteristics of tree imagery linked to mental disorders and evaluates their predictive efficacy. Following the PRISMA guidelines, we analyzed 42 studies involving 8552 participants from English and Chinese databases published between 1948 and 2024. The results showed that 24 specific characteristics significantly predicted mental disorders (*p* < 0.05), which were classified into five distinct categories: blackened out (e.g., blackened tree, odds ratio [OR] = 2.01), scribbled lines (e.g., weak lines, OR = 2.82), oddly shaped (e.g., flattened crown, OR = 3.10), no vitality (e.g., very small tree, OR = 3.93), and overly simple (e.g., simplified drawing, OR = 7.07). Furthermore, subgroup analyses indicated that features such as “blackened tree” (OR = 1.71), “no motion” (OR = 3.34), and “excessive separation” (OR = 2.77) were significantly associated with affective disorders, whereas the presence of “roots” (OR = 4.89) was uniquely associated with the thought‐disorder subgroup. Ultimately, tree imagery may offer a valuable nonverbal, adjunctive approach to screening for mental disorders, offering several statistically supported indicators that may effectively complement traditional assessment tools.

## 1. Introduction

Mental disorders represent a pressing global health challenge and impose a substantial burden on patients, families, and society as a whole [1]. Early screening and accurate identification are essential for mitigating the burden. While traditional rating scales are widely validated, they possess inherent limitations. For example, individuals with limited self‐awareness or cognitive impairments often struggle to provide accurate self‐assessments [2]. Furthermore, lengthy questionnaires can induce respondent fatigue and invite careless responses [3]. Most importantly, due to social expectations, subjects are likely to deliberately choose positive answers to hide their symptoms, thereby compromising the screening efficacy [4].

Projective drawing tests, such as the house‐tree‐person (HTP) test [5] and the tree drawing test (TDT) [6], offer a compelling nonverbal alternative. These nonverbal assessments use tree imagery to facilitate emotional and cognitive expression and rely less on verbal proficiency than many traditional tools [7, 8]. Empirical evidence highlights their potential for identifying conditions like depression, anxiety, and schizophrenia. For example, previous studies noted that blackened or lifeless trees were more frequently observed in adolescents with depressive symptoms [9], whereas distorted tree forms were more prevalent among young patients with schizophrenia [10]. These findings suggest that tree imagery may have value as a screening‐oriented, developmentally adaptable assessment approach.

Despite this potential, the broader clinical application of drawing tests remains hampered by inconsistent scoring criteria and subjective interpretation practices [11]. Such methodological variability in selecting and defining predictive indicators complicates cross‐study comparisons and undermines their standardized use in mental health settings. While previous systematic reviews have identified these challenges (e.g., [12, 13]), they have largely stopped short of providing a unified framework or conducting rigorous evaluations of the methodological quality of the primary literature. To address these gaps, the present systematic review and meta‐analysis synthesizes tree imagery characteristics associated with mental disorders while rigorously assessing the methodological quality of the included literature (utilizing the AHRQ criteria) to ensure a robust evidence base. Furthermore, to avoid the overgeneralization of psychodynamic interpretations, this study adopts a strictly data‐driven approach to evaluate the predictive validity. Specifically, we pose three key questions: (1) which tree imagery features are consistently employed to detect mental disorders? (2) How effectively do these features predict such conditions? (3) Do potentially distinct, statistically supported patterns emerge in predicting affective disorders (e.g., depression and anxiety) versus thought disorders (e.g., schizophrenia), while remaining cautious of over‐interpreting these as definitive disorder‐specific indicators? By addressing these questions, we aim to provide an objective, structured, and methodologically transparent foundation for the clinical use of tree imagery.

## 2. Methods

This systematic review and meta‐analysis was conducted in strict accordance with the PRISMA 2020 guidelines [14] and was registered in PROSPERO (CRD420251056682).

### 2.1. Literature Search

**Figure 1:**
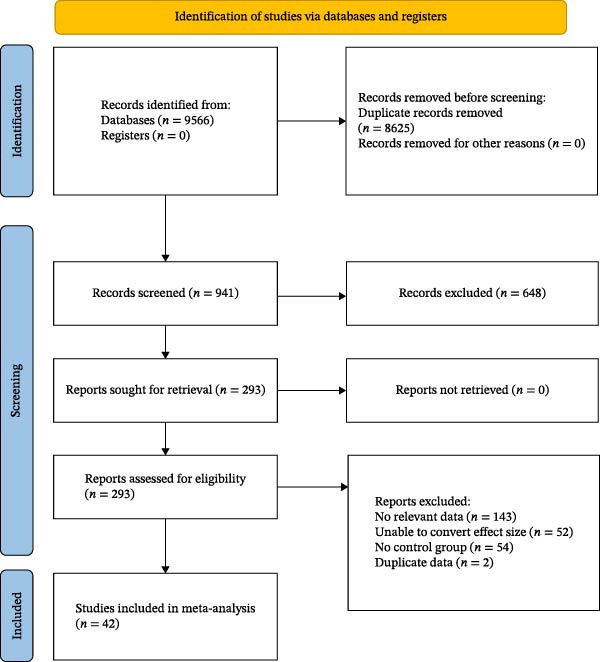
Preferred reporting items for systematic reviews and meta‐analyses (PRISMA) flow diagram of the article selection process.

We systematically searched English‐language databases (PubMed, Web of Science, and EBSCO) and Chinese‐language databases (China National Knowledge Infrastructure [CNKI], Wanfang, and Chinese Scientific Journal Database [VIP]) to identify studies published between January 1, 1948 and April 28, 2024. Search terms included “House Tree Person,” “Tree Drawing Test,” “Projective test,” and “Drawing test,” tailored to capture research relevant to mental health screening. Two authors independently screened 9566 records (titles, abstracts, and full texts), resolving any discrepancies through consensus discussion with a third author. A total of 42 studies (25 Chinese, 14 English, 2 Korean, and 1 Japanese) were ultimately included (Figure 1 provides detailed selection process).

### 2.2. Inclusion Criteria

Studies were considered eligible if they met the following criteria: (1) they investigated tree imagery in drawing tests related to mental disorders; (2) they defined mental disorders or clinically relevant psychopathological conditions using validated diagnostic criteria, clinical diagnoses, or established operational criteria in the original studies; (3) they compared participants with and without mental disorders; and (4) they reported extractable frequency data for specific tree imagery characteristics. Conversely, we excluded theoretical papers or review articles, studies lacking tree imagery analyses or adequate control groups, or studies with unconvertible effect sizes or duplicate data.

### 2.3. Quality Assessment

The methodological quality of the included studies was rigorously assessed using the AHRQ cross‐sectional study quality assessment form, an 11‐item checklist in which each item was scored as 0 (“no” or “unclear”) or 1 (“yes”). Studies were classified as high (8–11), medium (4–7), or low (0–3) quality. Two independent authors (Huibing Guo and Bin Feng) evaluated all 42 studies. Inter‐rater reliability was calculated for both the individual 11 items and the total quality score using Cohen’s *κ* coefficient. The overall agreement on the total score was almost perfect (*κ* = 0.85, 95% confidence interval [CI]: 0.78–0.92). Item‐level agreement ranged from substantial to almost perfect (*κ* = 0.72–0.91 across the 11 items). A total of 28 disagreements occurred across all item ratings (~6.1% of all 462 item ratings). All disagreements were resolved through consensus discussions between the two reviewers. When an initial consensus could not be reached (*n* = 3 cases), a third reviewer (Taolin Chen) was consulted for final adjudication. Detailed item‐by‐item scores for each study are provided in Supporting Information Table S1.

### 2.4. Data Coding

Two independent authors (Huibing Guo and Bin Feng) extracted and coded all data using a standardized form. To ensure consistency and comparability of effect sizes across heterogeneous studies (including those published in Chinese, Korean, or Japanese and using either the HTP test or the TDT), frequency data were first converted into 2 × 2 contingency tables for each tree imagery characteristic. A total of 45 characteristics that appeared in at least three studies were retained for analysis. Synonymous terms were unified (e.g., “blackened bark” vs. “blackened tree”), opposing features were inversely scored (e.g., “roots” vs. “no roots”), and conceptually similar items were merged into broader categories (e.g., “no flowers or grass” and “no decoration” were combined into “no additional decoration”). For the non‐English studies, characteristics were translated into English by a bilingual author (Zaiquan Dong), followed by back‐translation and independent review to ensure conceptual equivalence. All coding decisions were reached through iterative discussion and consensus between the two authors.

### 2.5. Statistical Analysis

Odds ratios (ORs) with 95% CIs were calculated to assess associations between tree imagery characteristics and mental disorders (*p* < 0.05, *Z*‐test). Heterogeneity was evaluated using the *Q*‐test (*p* ≤ 0.10) and *I* ^2^ statistic, applying a random‐effects model for *I* ^2^ ≥ 75% [15]. Publication bias was assessed using funnel plots, Rosenthal’s fail‐safe number (*N*_fs_ > 5*k* + 10), and the trim‐and‐fill method [16]. To avoid excessive fragmentation and to maintain readability, we summarized the quantitative findings in a tabular form rather than presenting a separate forest plot for each characteristic. Analyses were conducted using Comprehensive Meta‐Analysis 3.0.

## 3. Results

### 3.1. Study Characteristics

The meta‐analysis included 42 cross‐sectional studies comprising 8552 participants. Of these, 17 studies were published in English, Korean, or Japanese and 25 in Chinese, reflecting a strong representation of Asian youth populations. Collectively, these studies provided 957 independent effect sizes. Based on the quality assessment using the AHRQ cross‐sectional study quality assessment form, 30 studies were rated as high quality and 12 as medium quality, with no low‐quality studies included. The primary research predominantly focused on depression (*n* = 17), schizophrenia (*n* = 9), and anxiety (*n* = 3), with a substantial proportion conducted in youth samples. Key extracted data included author, publication year, drawing test type, sample size, group distribution (clinical vs. control), and disorder classification, are summarized in Table 1.

**Table 1:** Basic characteristics of the 42 included studies.

Author (year)	Test	Clinical group (*n*)	Control group (*n*)	Mental disorder	Quality score
Eisel (1978) [17]	HTP	69	69	Schizophrenia	9
Fukunishi (2002) [18]	HTP	50	142	Alexithymia	8
Guo (2022) [19]	HTP	55	112	Depression	9
Inadomi (2003) [20]	TDT	20	53	Schizophrenia	8
Kaneda (2010) [21]	TDT	202	113	Schizophrenia	8
Ki (2016) [22]	TDT	77	281	Depression	8
Kim (2021) [23]	DTF‐D	32	63	Depression	8
Kirchner (1974) [24]	HTP	49	146	Substance addiction disorder	5
Koide (1992) [25]	HTP	16	110	Organic mental disorder	6
Kwark (2010) [26]	HTP	50	50	Schizophrenia	6
Lee (2019) [27]	HTP	23	163	Depression	7
Lee (2020) [28]	HTP	60	126	Substance addiction disorder	7
Murayama (2016) [29]	TDT	14	145	Depression	8
Robens (2019) [30]	TDT	64	67	Cognitive disorder	8
Sheng (2019) [31]	HTP	27	140	Anxiety	7
Yang (2019) [32]	HTP	57	110	Depression	10
Zhou (2019) [10]	HTP	17	22	Schizophrenia	8
Chen (2015) [33]	HTP	30	30	Schizophrenia	9
Chen (2015) [34]	HTP	38	524	Personality disorder	10
Deng (2014) [35]	HTP	32	32	Schizophrenia	9
Deng (2017) [36]	HTP	30	30	Depression	6
Gao (2019) [37]	TDT	212	241	Depression	9
Huang (2016) [38]	HTP	5	5	Autism	8
Jin (2020) [39]	TDT	63	97	Depression	7
Li (2016) [40]	HTP	30	35	Depression	6
Li (2021) [13]	HTP	30	30	Depression	8
Li (2020) [41]	HTP	190	134	Anxiety	10
Li (2014) [42]	HTP	35	70	Autism	9
Ning (2015) [43]	HTP	148	528	Depression	9
Tang (2017) [44]	TDT	53	85	Depression	4
Wang (2007) [45]	HTP	25	30	Mental disorder	8
Wang (2017) [46]	HTP	74	103	Anxiety	7
Xiang (2020) [47]	HTP	22	336	ADHD	8
Xiang (2020) [48]	HTP	68	290	Depression	8
Xie (1994) [49]	HTP	110	110	Schizophrenia	8
Yan (2012) [9]	TDT	70	79	Depression	8
Yan (2014) [50]	HTP	277	263	Depression	10
Zhang (2019) [51]	TDT	60	60	Depression	6
Zhao (2015) [52]	HTP	37	133	Somatization disorder	9
Zhou (2021) [53]	HTP	100	100	Rumination	10
Zhu (2011) [54]	HTP	59	53	PTSD	10
Zhu (2020) [55]	HTP	140	422	Personality disorder	10

*Note*: Author (year), the name of the first author with publication year. Quality scores were assessed with the AHRQ cross‐sectional study quality assessment form (0–11 points; Supporting Table S1 provides item‐by‐item scores).

Abbreviations: ADHD, attention‐deficit/hyperactivity disorder; DTF‐D, drawing test form for depression; HTP, house‐tree‐person test; PTSD, post‐traumatic stress disorder; TDT, tree drawing test.

### 3.2. Predictive Characteristics

Across the 42 studies, 358 unique drawing characteristics were initially identified, with 166 specifically pertaining to tree imagery. After filtering by frequency, 45 characteristics that appeared in at least three studies were analyzed for predictive validity. Of these, 24 demonstrated statistically significant associations with mental disorders (*p* < 0.05) and were categorized into five thematic groups (Table 2).

**Table 2:** Predictive effects of tree imagery characteristics on mental disorders.

Category	Drawing characteristics	*k*	Heterogeneity		OR	95% CI	*p*	*N*_fs_
			*Q* (*p*)	*I* ^2^ (%)				
Blackened out	Blackened tree	17	<0.001	79.20	2.01	1.29, 3.09	0.002	131
	Blackened non‐tree elements	5	0.027	63.46	2.49	1.68, 3.7	<0.001	30
	Shadow	3	0.411	0.00	2.88	1.36, 6.11	0.006	5
Scribbled lines	Scribbled lines of tree	5	0.088	50.68	2.84	1.9, 4.23	<0.001	35
	Weak or intermittent tree lines	7	0.252	23.24	2.82	2.14, 3.73	<0.001	97
	Weak or intermittent other lines	9	0.202	27.23	2.58	1.91, 3.48	<0.001	95
	Trembling lines of tree	4	0.101	51.81	2.47	1.46, 4.2	0.001	6
Oddly shaped	Disproportionate tree	3	<0.001	87.31	3.15	1.12, 8.88	0.030	28
	Right leaning tree	3	0.015	74.27	1.79	1.25, 2.56	0.002	35
	Flattened crown	7	0.004	68.34	3.1	2.38, 4.03	<0.001	109
	Closed trunk	3	0.536	0.00	2.51	1.61, 3.89	<0.001	11
	Very long trunk	3	0.203	37.35	2.31	1.43, 3.75	0.001	8
	Sharp branch	6	0.007	68.60	2.35	1.6, 3.46	<0.001	26
	Roots	7	<0.001	76.18	2.23	1.23, 4.02	0.008	22
No vitality	Very small tree	11	0.050	45.45	3.93	2.99, 5.17	<0.001	248
	Dead tree	7	0.013	62.78	3.47	2.33, 5.17	<0.001	65
	Truncated tree	7	<0.001	79.83	3.33	1.23, 9.02	0.018	37
	Broken branches	3	0.145	48.13	2.14	1.13, 4.04	0.019	5
	Sagging crowns	3	0.167	44.14	2.49	1.05, 5.93	0.039	10
	No motion	5	0.201	37.60	4.03	2.74, 5.92	<0.001	35
Overly simple	Simplified drawing	12	<0.001	82.55	7.07	3.63, 13.75	<0.001	507
	Small drawing size	5	0.298	18.34	5.76	3.42, 9.69	<0.001	51
	Excessive separation	8	<0.001	82.62	3.24	1.79, 5.87	<0.001	142
	No additional decoration	20	<0.001	91.96	2.62	1.46, 4.7	0.001	242

*Note*: *k*, number of studies; *N*_fs_, Rosenthal’s fail‐safe number. All characteristics shown were statistically significant (*p* < 0.05).

Abbreviations: 95% CI, 95% confidence interval; OR, odds ratio.

The “blackened out” category included features such as “blackened tree” (OR = 2.01, 95% CI: 1.29–3.09, *p* = 0.002) which may potentially indicate negative emotional states. “Scribbled lines” included “weak or intermittent tree lines” (OR = 2.82, 95% CI: 2.14–3.73, *p* < 0.001), reflecting low mental energy. The “oddly shaped” category included “flattened crown” (OR = 3.10, 95% CI: 2.38–4.03, *p* < 0.001), suggesting distorted perceptions. The “no vitality” category featured “very small tree” (OR = 3.93, 95% CI: 2.99–5.17, *p* < 0.001), potentially signaling hopelessness, while “overly simple” highlighted “simplified drawing” (OR = 7.07, 95% CI: 3.63–13.75, *p* < 0.001), indicating psychological withdrawal or disengagement.

### 3.3. Subgroup Analysis

To explore potential disorder‐specific patterns, mental disorders were broadly classified into affective (e.g., depression, anxiety, and PTSD) and thought (e.g., schizophrenia, obsessive–compulsive disorder, and schizotypal personality disorder) categories [56, 57]. We analyzed 15 characteristics that appeared in at least three studies within their respective subgroups (results are detailed in Table 3).

**Table 3:** Subgroup analysis by disorder type (affective‐ vs. thought‐disorders).

Category	Drawing characteristics	Type	*k*	Heterogeneity		OR	95% CI	*p*
				*Q* (*p*)	*I* ^2^ (%)			
Affect‐specific indicator	Blackened tree	AD	14	<0.001	67.87	1.71	1.41, 2.08	**<0.001**
		TD	3	<0.001	91.07	1.24	0.22, 6.97	0.807
	No motion	AD	3	<0.001	86.99	3.34	1.22, 9.16	**0.019**
		TD	2	0.001	91.06	2.63	0.63, 11.02	0.185
	Excessive separation	AD	4	<0.001	87.28	2.77	1.15, 6.68	**0.023**
		TD	3	0.001	85.47	7.51	0.72, 78.46	0.092
Thought‐specific indicator	Roots	AD	4	0.244	28.04	1.59	0.93, 2.47	0.094
		TD	2	<0.001	87.01	4.89	2.96, 8.08	**<0.001**
Mental disorder co‐indicator	Weak or intermittent tree lines	AD	7	0.545	0.00	2.28	1.66, 3.13	**<0.001**
		TD	2	0.399	0.00	7.01	2.84, 17.3	**<0.001**
	No additional decoration	AD	13	<0.001	91.83	2.57	1.41, 4.67	**0.002**
		TD	4	0.002	77.57	16.33	5.01, 53.2	**<0.001**
	Simplified drawing	AD	5	<0.001	89.39	8.57	2.54, 28.94	**0.001**
		TD	5	0.001	78.73	4.76	1.77, 12.8	**0.002**
	Small drawing size	AD	3	0.188	40.16	5.16	2.61, 10.19	**<0.001**
		TD	2	0.252	23.94	6.72	3, 15.08	**<0.001**
	Very small tree	AD	8	0.014	60.17	4.14	2.99, 5.74	**<0.001**
		TD	3	0.813	0.00	3.46	2.08, 5.75	<0.001

*Note*: Bold *p*‐values indicate statistical significance (*p* < 0.05).

Abbreviations: AD, affective disorder; TD, thought disorder.

For affective disorders, the features “blackened tree” (OR = 1.71, 95% CI: 1.41–2.08, *p* < 0.001), “no motion” (OR = 3.34, 95% CI: 1.22–9.16, *p* = 0.019), and “excessive separation” (OR = 2.77, 95% CI: 1.15–6.68, *p* = 0.023) demonstrated significant associations in the affective‐disorder subgroup. In contrast, the presence of “roots” (OR = 4.89, 95% CI: 2.96–8.08, *p* < 0.001) exhibited a significant association uniquely within the thought‐disorder subgroup, though this preliminary finding should be interpreted cautiously due to the limited number of contributing studies. Furthermore, nonspecific predictors such as “weak or intermittent tree lines” (affective: OR = 2.28, *p* < 0.001; thought: OR = 7.01, *p* < 0.001) and “very small tree” (affective: OR = 4.14, *p* < 0.001; thought: OR = 3.46, *p* < 0.001) displayed significant associations across both the affective and thought disorder subgroups.

### 3.4. Publication Bias

**Figure 2:**
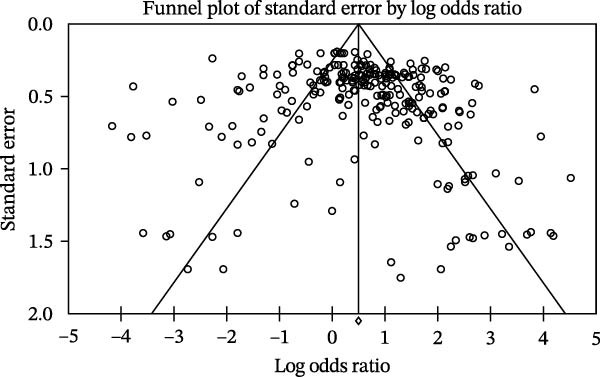
Funnel plot assessing publication bias for tree imagery characteristics and mental disorders.

Publication bias was evaluated using funnel plots, Rosenthal’s fail‐safe number (*N*_fs_), and the trim‐and‐fill method. As seen in Figure 2, funnel plots were broadly symmetrical overall, indicating no robust evidence of a substantial publication bias. For the majority of characteristics, the *N*_fs_ exceeded the conventional threshold of 5*k* + 10 (e.g., “simplified drawing,” *N*_fs_ = 507, *k* = 12), whereas characteristics supported by fewer studies warrant more cautious interpretation. Trim‐and‐fill analyses generally yielded adjusted effect estimates in the same direction as the original computations (e.g., “truncated tree,” adjusted OR = 2.01 vs. original OR = 3.33), although the magnitude of some pooled effects was attenuated (results detailed in Table 4). Overall, the publication‐bias assessments support the relative robustness and stability of the primary findings.

**Table 4:** Assessment of publication bias using the trim‐and‐fill method.

Drawing characteristics	*k*	Original OR	Adjusted OR	95% CI
Blackened non‐tree elements	1	2.49	2.34	1.58, 3.46
Shadow	0	2.88	2.88	1.36, 6.11
Trembling lines of tree	0	2.47	2.47	1.46, 4.2
Closed trunk	2	2.51	2.07	1.42, 3.02
Very long trunk	0	2.31	2.31	1.43, 3.75
Sharp branch	1	2.35	2.18	1.51, 3.18
Roots	0	2.23	2.23	1.23, 4.02
Truncated tree	2	3.33	2.01	0.75, 5.36
Broken branches	2	2.14	1.67	0.92, 3.04
Sagging crowns	0	2.49	2.49	1.05, 5.93

*Note*: Adjusted OR, pooled odds ratio after trim‐and‐fill correction for potential publication bias; *k*, number of potentially missing studies imputed by the trim‐and‐fill procedure for that characteristic; original OR, pooled odds ratio before trim‐and‐fill adjustment.

## 4. Discussion

The primary objective of this systematic review and meta‐analysis was to evaluate the predictive validity of tree imagery in projective drawing tests and to establish a unified, evidence‐based framework for mental health screening. By synthesizing data from 42 methodologically robust cross‐sectional studies encompassing 8552 participants, we identified 24 statistically significant characteristics. Innovatively, we adopted a strictly data‐driven approach to organize these indicators into a novel five‐category framework: blackened out, scribbled lines, oddly shaped, no vitality, and overly simple. Furthermore, subgroup analyses uncovered detailed, provisional patterns differentiating psychiatric conditions: features such as the “blackened tree,” “no motion,” and “excessive separation” demonstrated significant associations specifically with affective disorders, whereas the presence of “roots” emerged as a distinctly associated pattern within the thought‐disorder subgroup. We also identified nonspecific markers, such as “weak or intermittent tree lines” and “very small tree,” which were shared across both domains, likely reflecting general psychological distress rather than a singular psychiatric condition. Ultimately, this study underscores the clinical significance of tree imagery as a nonverbal, adjunctive screening tool. By circumventing the inherent limitations of conventional self‐report scales, such as social desirability biases and verbal constraints, these statistically supported indicators provide clinicians with a valuable complementary method for identifying early signs of mental disorders.

### 4.1. Predictive Effects of Tree Imagery on Mental Disorders

While tree imagery has traditionally been interpreted through a psychodynamic lens [58, 59], our five‐category framework—blackened out, scribbled lines, oddly shaped, no vitality, and overly simple—offers an empirically grounded, data‐driven structure to organize tree‐imagery characteristics associated with mental disorders.

The “blackened out” category (e.g., blackened tree and shadows) may be interpreted as reflecting negative valence or less consciously integrated aspects of the psychological experience. In Jung theory, the “shadow” refers to the darker, less accepted aspects of personality, often described as emotionally charged and resistant to moral control [60]. These features were significantly associated with mental disorders in the present meta‐analysis and may have potential screening relevance, particularly when interpreted alongside other clinical information.

Similarly, “scribbled lines” (e.g., weak lines, prevalence: 40.4%; [32]) may be associated with diminished mental energy and emotional instability, consistent with psychoanalytic views [61]. These indicators may provide clinically relevant descriptive cues, but their role in identifying emotional regulation difficulties and informing targeted interventions requires further prospective validation.

“Oddly shaped” trees (e.g., flattened crowns and sharp branches) may be interpreted as reflecting atypical or strained forms of expression [51]. Flattened crowns and sharp branches may represent visually salient features reported in affective‐ or thought‐disorder samples, but their disorder‐specific psychological interpretation remains provisional [33]. Importantly, “no vitality” features (e.g., small and dead trees) may be associated with reduced vitality, hopelessness, or psychological burden, aligning with prior interpretive literature [6, 62]. Some prior studies have discussed possible links between such features and severe distress; however, the present meta‐analysis does not permit direct inference about the suicide risk or other specific clinical outcomes [18]. Finally, “overly simple” drawings (e.g., minimal decoration) may be associated with withdrawal, which is common in depression and autism [26, 63].

### 4.2. Subgroup Analysis

Our subgroup findings suggest potentially different association patterns, with possible implications for theory development and future clinical validation. In the current subgroup analyses, “blackened tree,” “no motion,” and “excessive separation” showed significant associations in the affective‐disorder subgroup. These features were statistically significant in the affective‐disorder subgroup in the present dataset [46], whereas corresponding associations did not reach significance in the thought‐disorder subgroup [49]. Conversely, the presence of “roots” showed a statistically significant association in the thought‐disorder subgroup, which may warrant further attention in future studies. However, given the small number of studies contributing to this subgroup, this finding should be interpreted cautiously and requires replication in larger samples. Similarly, while “simplified drawing” showed significant associations across subgroup types, its disorder‐specific interpretation remains preliminary. Furthermore, common indicators—“weak lines,” “no decoration,” “simplified drawing,” “small size,” and “very small tree”—appeared across subgroup types and may represent relatively nonspecific correlates of low energy, withdrawal, or reduced expressive elaboration [32]. These shared markers may provide useful candidates for future screening‐oriented validation.

### 4.3. Theoretical Contributions and Clinical Implications

This study contributes to psychodynamic and projective‐test research by integrating empirical findings into a five‐category framework and helping to organize previously heterogeneous interpretive patterns [11]. The 24 statistically supported characteristics, including several subgroup‐associated patterns, help organize empirically supported drawing features into a more structured framework, thereby improving the transparency of projective‐test research.

Because tree imagery relies less heavily on verbal report, it may be particularly useful in contexts where developmental or linguistic factors limit conventional self‐report assessment [64]. Features like “no motion” or “small trees” may indicate distress and could help flag individuals who warrant further clinical evaluation. With appropriate training and standardized scoring guidance, these indicators may support broader screening use in clinical and community settings. Combining drawing tests with traditional scales may enrich preliminary screening across patient groups, particularly among individuals with limited expressive capacity.

### 4.4. Limitations

Several limitations should be acknowledged. First, many included studies provided limited demographic information (e.g., gender and exact age distributions), which restricted subgroup analyses by participant characteristics. Second, the predominance of Asian populations—particularly Chinese youth samples—limits the cross‐cultural generalizability of our findings. Cultural symbolic conventions, artistic habits, and diverse emotional expression styles may significantly influence how tree‐imagery characteristics are produced and interpreted, necessitating cautious application in other cultural contexts. Third, several characteristics (e.g., “shadows”) were supported by only a small number of studies and showed substantial heterogeneity, which may have inflated or destabilized some pooled estimates. Finally, the binary affective‐versus‐thought‐disorder classification does not capture the full range of psychiatric and developmental conditions represented in the literature. Future studies should include more diverse populations, richer demographic data, and broader validation across cultural and diagnostic contexts.

## 5. Conclusion

This meta‐analysis affirms the value of tree imagery in projective drawing tests as a viable nonverbal adjunctive tool for mental health screening. By adopting a strictly data‐driven approach, we identified 24 statistically significant characteristics organized into a comprehensive five‐category framework: blackened out, scribbled lines, oddly shaped, no vitality, and overly simple. Furthermore, the synthesis of both common nonspecific indicators and provisional subgroup‐related patterns provides a structured foundation that can inform future standardization efforts. While these drawing‐based features offer clinicians valuable supporting information when used alongside traditional rating scales, their clinical application must remain appropriately cautious. Given the current predominance of Asian youth samples in the literature, further prospective studies across diverse cultural contexts and broader demographic populations are essential to validate their cross‐cultural generalizability, incremental diagnostic accuracy, and practical impact on intervention decisions.

## Funding

This study was supported by the National Natural Science Foundation of China (Grant 82572350) and the Scientific Research Starting Foundation for Young Teachers, Sichuan University (Special Project for Ideological and Political Education Instructors) (Grant sksz202505).

## Disclosure

An earlier version of this manuscript was published as a preprint: Huibing Guo, Bin Feng, Tiantian Liu, Ruopeng Zhao, Huiyong Fan, Zaiquan Dong, Qiyong Gong, and Taolin Chen. Tree Imagery in Drawing Tests for Screening Mental Disorders: A Systematic Review and Meta‐Analysis. Research Square https://doi.org/10.21203/rs.3.rs-4584440/v1.

## Ethics Statement

The current study used publicly available data and was, therefore, exempted from ethics approval and written informed consent procedures.

## Conflicts of Interest

The authors declare no conflicts of interest.

## Supporting Information

Additional supporting information can be found online in the Supporting Information section.

## Supporting information


**Supporting Information** The supporting material for this article includes a detailed quality assessment of all included studies. Table S1 presents the item‐by‐item scores based on the AHRQ cross‐sectional study quality assessment form (0–11 points) for each of the 42 studies.

## Data Availability

The data that support the findings of this study are available upon request from the corresponding author. The data are not publicly available due to privacy or ethical restrictions.
