# Supplementary material for: Tree Imagery in Drawing Tests for Screening Mental Disorders: A Systematic Review and Meta‐Analysis
Source: Depress Anxiety. 2026 Jul 11;2026:9571222. doi: 10.1155/da/9571222 (PMC13355497; doi:10.1155/da/9571222)
Supplement: Supplementary file 1 — Supporting Information The supporting material for this article includes a detailed quality assessment of all included studies. Table S1 presents the item‐by‐item scores based on the AHRQ cross‐sectional study quality assessment form (0–11 points) for each of the 42 studies. [file DA-2026-9571222-s001.docx]

Table S1. Detailed AHRQ quality assessment of the included studies

| Author (Year) | 1 | 2 | 3 | 4 | 5 | 6 | 7 | 8 | 9 | 10 | 11 | Total | Quality |
| --- | --- | --- | --- | --- | --- | --- | --- | --- | --- | --- | --- | --- | --- |
| Eisel (1978) | 1 | 1 | 1 | 1 | 1 | 1 | 1 | 1 | 0 | 1 | 0 | 9 | High |
| Fukunishi (2002) | 1 | 1 | 1 | 1 | 1 | 1 | 1 | 1 | 0 | 0 | 0 | 8 | High |
| Guo (2022) | 1 | 1 | 1 | 1 | 1 | 1 | 1 | 1 | 0 | 1 | 0 | 9 | High |
| Inadomi (2003) | 1 | 1 | 0 | 1 | 1 | 1 | 0 | 1 | 0 | 1 | 1 | 8 | High |
| Kaneda (2010) | 1 | 1 | 0 | 1 | 1 | 1 | 0 | 1 | 0 | 1 | 1 | 8 | High |
| Ki (2016) | 1 | 1 | 0 | 1 | 1 | 1 | 0 | 1 | 0 | 1 | 1 | 8 | High |
| Kim (2021) | 1 | 1 | 1 | 1 | 0 | 1 | 0 | 1 | 0 | 1 | 1 | 8 | High |
| Kirchner (1974) | 1 | 1 | 0 | 1 | 1 | 1 | 0 | 0 | 0 | 0 | 0 | 5 | Med. |
| Koide (1992) | 1 | 1 | 0 | 1 | 1 | 1 | 0 | 1 | 0 | 0 | 0 | 6 | Med. |
| Kwark (2010) | 1 | 1 | 0 | 1 | 1 | 1 | 0 | 1 | 0 | 0 | 0 | 6 | Med. |
| Lee (2019) | 1 | 1 | 1 | 1 | 0 | 1 | 1 | 1 | 0 | 0 | 0 | 7 | Med. |
| Lee (2020) | 1 | 1 | 1 | 1 | 0 | 1 | 1 | 1 | 0 | 0 | 0 | 7 | Med. |
| Murayama (2016) | 1 | 1 | 0 | 1 | 0 | 1 | 1 | 1 | 0 | 1 | 1 | 8 | High |
| Robens (2019) | 1 | 1 | 0 | 1 | 1 | 1 | 0 | 1 | 0 | 1 | 1 | 8 | High |
| Sheng (2019) | 1 | 1 | 1 | 1 | 0 | 1 | 1 | 1 | 0 | 0 | 0 | 7 | Med. |
| Yang (2019) | 1 | 1 | 1 | 1 | 1 | 1 | 1 | 1 | 1 | 1 | 0 | 10 | High |
| Zhou (2019) | 1 | 1 | 1 | 1 | 1 | 1 | 1 | 1 | 0 | 0 | 0 | 8 | High |
| Chen LY (2015) | 1 | 1 | 1 | 1 | 1 | 1 | 1 | 1 | 0 | 1 | 0 | 9 | High |
| Chen T (2015) | 1 | 1 | 1 | 1 | 1 | 1 | 1 | 1 | 1 | 0 | 1 | 10 | High |
| Deng (2014) | 1 | 1 | 1 | 1 | 1 | 1 | 1 | 1 | 0 | 1 | 0 | 9 | High |
| Deng (2017) | 1 | 1 | 0 | 0 | 0 | 1 | 1 | 1 | 0 | 0 | 1 | 6 | Med. |
| Gao (2019) | 1 | 1 | 1 | 1 | 1 | 1 | 1 | 1 | 0 | 1 | 0 | 9 | High |
| Huang (2016) | 1 | 1 | 0 | 1 | 1 | 1 | 0 | 1 | 0 | 1 | 1 | 8 | High |
| Jin (2020) | 1 | 1 | 1 | 1 | 0 | 1 | 0 | 1 | 0 | 0 | 1 | 7 | Med. |
| Li (2016) | 1 | 1 | 0 | 1 | 0 | 1 | 1 | 0 | 0 | 0 | 1 | 6 | Med. |
| Li (2021) | 1 | 1 | 0 | 1 | 1 | 1 | 0 | 1 | 0 | 1 | 1 | 8 | High |
| Li (2020) | 1 | 1 | 1 | 1 | 1 | 1 | 1 | 1 | 0 | 1 | 1 | 10 | High |
| Li (2014) | 1 | 1 | 1 | 1 | 0 | 1 | 1 | 1 | 0 | 1 | 1 | 9 | High |
| Ning (2015) | 1 | 1 | 1 | 1 | 1 | 1 | 1 | 1 | 0 | 1 | 0 | 9 | High |
| Tang (2017) | 1 | 0 | 0 | 1 | 0 | 0 | 0 | 0 | 0 | 1 | 1 | 4 | Med. |
| Wang (2007) | 1 | 1 | 1 | 1 | 0 | 1 | 0 | 1 | 0 | 1 | 1 | 8 | High |
| Wang (2017) | 1 | 1 | 1 | 1 | 0 | 1 | 0 | 0 | 0 | 1 | 1 | 7 | Med. |
| Xiang (2020a) | 1 | 1 | 0 | 1 | 0 | 0 | 1 | 1 | 1 | 1 | 1 | 8 | High |
| Xiang (2020b) | 1 | 1 | 0 | 1 | 0 | 0 | 1 | 1 | 1 | 1 | 1 | 8 | High |
| Xie (1994) | 1 | 1 | 0 | 1 | 1 | 1 | 1 | 1 | 1 | 0 | 0 | 8 | High |
| Yan (2012) | 1 | 1 | 1 | 1 | 1 | 1 | 0 | 1 | 0 | 0 | 1 | 8 | High |
| Yan (2014) | 1 | 1 | 0 | 1 | 1 | 1 | 1 | 1 | 1 | 1 | 1 | 10 | High |
| Zhang (2019) | 1 | 1 | 0 | 1 | 1 | 1 | 0 | 1 | 0 | 0 | 0 | 6 | Med. |
| Zhao (2015) | 1 | 1 | 1 | 1 | 0 | 1 | 1 | 1 | 0 | 1 | 1 | 9 | High |
| Zhou (2021) | 1 | 1 | 1 | 1 | 1 | 1 | 1 | 1 | 0 | 1 | 1 | 10 | High |
| Zhu (2011) | 1 | 1 | 1 | 1 | 1 | 1 | 1 | 1 | 1 | 0 | 1 | 10 | High |
| Zhu (2020) | 1 | 1 | 1 | 1 | 1 | 1 | 1 | 1 | 1 | 0 | 1 | 10 | High |

*Note*: AHRQ quality was assessed using the 11-item checklist for cross-sectional/observational studies. Each item was scored as 1 = Yes and 0 = No/Unclear. Total scores were categorized as low (0–3), moderate (4–7), and high (8–11). Unreported items were conservatively scored as 0.

**AHRQ 11-item checklist (exact items used)**

Q1: Define the source of information (survey, record review, etc.)

Q2: List inclusion and exclusion criteria for exposed and unexposed subjects (cases and controls) or refer to previous publications

Q3: Indicate the time period used for identifying patients

Q4: Indicate whether subjects were consecutive if not population-based

Q5: Indicate if evaluators of subjective components of the study were masked to other aspects of participants’ status

Q6: Describe any assessments undertaken for quality assurance purposes

Q7: Explain any patient exclusions from analysis

Q8: Describe how confounding was assessed and/or controlled

Q9: Explain how missing data were handled

Q10: Summarize response rates and completeness of data collection

Q11: Clarify follow-up, or indicate that follow-up was not applicable to the study design
